# The Influence of the Sympathetic Nervous System on Cardiometabolic Health in Response to Weight Gain or Weight Loss

**DOI:** 10.3390/metabo15050286

**Published:** 2025-04-23

**Authors:** Gavin W. Lambert, Mariya Patel, Elisabeth A. Lambert

**Affiliations:** 1 School of Health Sciences and Iverson Health Innovation Research Institute, Swinburne University of Technology, Hawthorn, VIC 3122, Australia; mariyapatel@swin.edu.au (M.P.); elisabethlambert@swin.edu.au (E.A.L.); 2 Medical Technology Victoria (MedTechVic) Research Hub, Hawthorn, VIC 3122, Australia

**Keywords:** obesity, body weight, autonomic nervous system, brain pathways, cachexia, anorexia, cardiovascular risk, diabetes, noradrenaline

## Abstract

Alterations in sympathetic nervous activity are evident in response to changes in body weight. Sympathetic nervous activity and sympathetic responses to weight change are regionalized, with alterations in end organ function dependent on the changes occurring in the brain regulatory pathways invoked and in the effector organs engaged. The obesity-induced activation of the sympathetic nervous system likely contributes to the initiation and worsening of cardiometabolic risk factors, including elevated blood pressure, cardiac dysfunction, dyslipidaemia, increased fasting blood glucose, insulin resistance, and non-alcoholic steatohepatitis. Unintended weight loss, as occurs in cachexia, is driven, at least in part, by the activation of sympathetic nervous-stimulated thermogenesis. The complexity of sympathetic nervous regulation renders the use of global measures of sympathetic activity problematic and the development of targeted therapies difficult, but these are not without promise or precedent. Knowledge of the central and peripheral pathways involved in sympathetic nervous regulation has opened up opportunities for pharmacological, surgical, and device-based approaches to mitigating the burden of disease development and progression. In this narrative review, we elaborate on sympathetic activity in response to changes in body weight, the brain pathways involved, and the cardiovascular and metabolic risks associated with perturbations in regional sympathetic activity.

## 1. Introduction

When viewed as a continuous variable, being at each end of the body weight spectrum confers an elevated risk of cardiometabolic disease development. Overweight and obesity are well recognised as conferring an increased risk for hypertension [1], diabetes [2], and cardiovascular disease [3], with the elevated risk of disease development being driven by the accumulation of body fat and fat distribution rather than increased body mass index (BMI) per se [4]. The excess adipose tissue in obese individuals produces adipokines that can act centrally and inflammatory cytokines that promote endothelial dysfunction, vascular inflammation, and oxidative stress, further exacerbating cardiometabolic risk [5,6,7]. The relationship between adiposity and cardiovascular risk is, however, not linear and better described as U-shaped, given that large population studies have indicated that being underweight is also linked with increased relative risk of stroke, myocardial infarction, and coronary artery disease [8,9,10]. Unintentional weight loss seen in clinical conditions, such as anorexia nervosa and cachexia, is associated with cardiovascular and metabolic complications.

While the pathophysiological mechanisms underlying the development of metabolic syndrome and the generation of obesity-related illness are complex and extend beyond a sedentary lifestyle, poor diet, and genetic predisposition, it is clear from clinical and preclinical observations that the sympathetic nervous system is important in the generation of obesity-related illness. Chronic sympathetic activation in obesity leads to high blood pressure, causes metabolic dysregulation, and contributes to the development of left ventricular hypertrophy, arterial stiffness, and endothelial dysfunction, all of which are independent risk factors for cardiovascular disease. The role of the sympathetic nervous system in underweight-related illness is less clear, largely due to the limited investigations at the stages of disease development and recovery.

**Figure 1:**
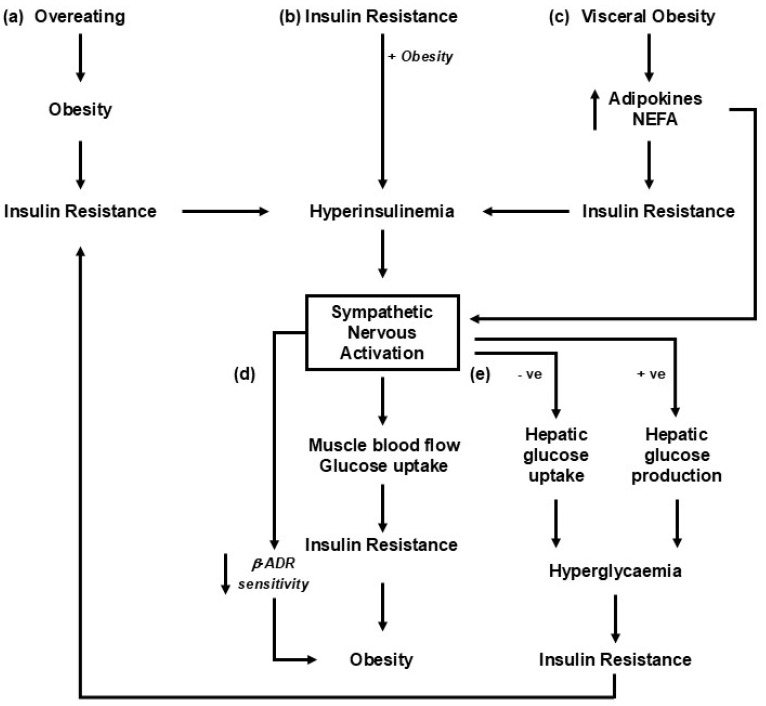
Putative mechanisms linking changes in weight with sympathetic nervous activation. The roles of insulin and hyperinsulinemia are likely important, given their critical role in metabolic syndrome development [18]. The pathways represented share a common role in engaging the liver and pancreas, leading to the development of hyperinsulinemia and insulin resistance. (**a**) Sympathetic nervous activation occurs as an adaptive response to overeating [19]. (**b**) The precipitating factor in the development of obesity-related illness is insulin resistance, driving hyperinsulinemia, sympathetic activation, and an increase in blood pressure [20]. (**c**) Increments in visceral fat result in increased release of adipokines, including leptin and non-esterified fatty acids (NEFAs), into circulation, which leads to insulin resistance and sympathetic nervous activation [21]. (**d**) Insulin resistance and the development of obesity-related illness are driven primarily by sympathetic nervous activation and increased norepinephrine release [22]. (**e**) An alteration in sympathetic drive to the liver with weight gain or diet change influences the hepatic uptake of glucose and impacts the development of insulin resistance [23,24].

The regionalized nature of sympathetic nervous activity and the differing short- and long-term regulatory responses that may occur mean that developing a unified in toto hypothesis of sympathetic regulation in response to changes in body weight is difficult. While obesity may arise due to a reduction in sympathetic-mediated thermogenesis [11], perhaps due to altered activity in the hypothalamus [12] or reduced beta-adrenergic signalling in the adipose tissue [13,14], on balance, clinical and experimental data also point to weight gain and obesity being associated with region-specific sympathetic nervous activation, with concomitant changes in physiological markers such as blood pressure, insulin resistance, and kidney function. Intentional weight loss is typically accompanied by the reverse. The mechanism(s) responsible have not been unequivocally characterised but likely involve interactions between multiple metabolic and hemodynamic regulatory pathways (Figure 1). Clearly, even small increments in weight are associated with sympathetic activation [15] and elevated plasma noradrenaline and altered reflex sympathetic responses have been shown to predict subsequent weight gain and the development of insulin resistance [16,17]. In this review, we examine the association of sympathetic nervous activity with changes in body weight. We focus on the brain pathways involved in sympathetic regulation and examine regional patterns in sympathetic activity and their relation with cardiometabolic risk associated with changes in weight.

## 2. Assessing Sympathetic Nervous Activity

**Figure 2:**
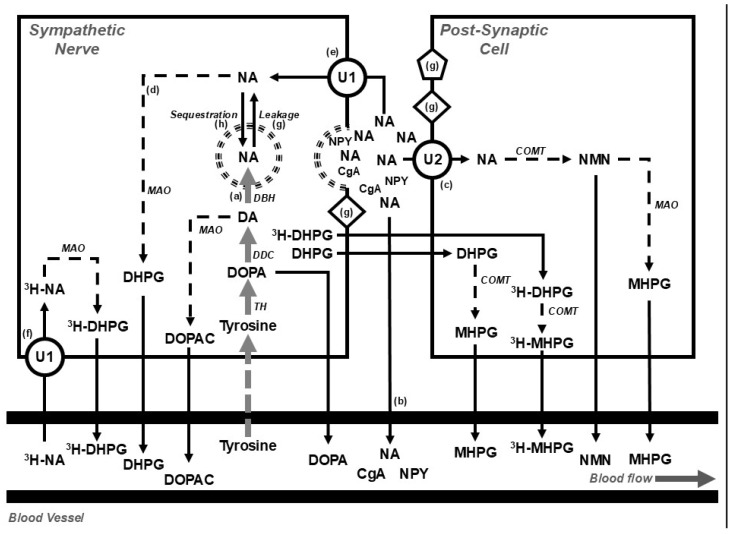
Diagram showing (top) the disposition of noradrenaline in postganglionic sympathetic nerve endings and post-synaptic cells (e.g., blood vessels, muscle cells, or cardiac myocytes) and (bottom) overflow into the systemic circulation under basal conditions and during the steady-state infusion of a tracer dose of tritium-labelled noradrenaline (^3^H-NA). The use of tritium enables the calculation of the rate of noradrenaline clearance from, and spillover into, plasma. At steady state, the rate of noradrenaline synthesis (**a**) equals the rate of noradrenaline turnover; where the noradrenaline turnover is equal to the sum of the rates of the spillover of noradrenaline into plasma (**b**), the extraneuronal uptake and metabolism of noradrenaline (**c**), and the intraneuronal metabolism of noradrenaline (**d**). The availability of noradrenaline for intraneuronal metabolism is determined by the balance between the entry of noradrenaline into the axoplasm, via the processes of reuptake (**e**) and leakage (**g**), and removal from the axoplasm by vesicular sequestration. Circulating ^3^H-NA may be extracted from plasma via the process of neuronal uptake (uptake 1; U1) and metabolised intraneuronally (**f**) or extraneuronally (U2). Following an influx of calcium, storage vesicles migrate to the nerve ending and release their contents. The contents include noradrenaline and other vesicular constituents, including neuropeptide y (NPY) and chromogranin a (CgA). Compounds released from the storage vesicles can act on pre- or post-neuronal receptors (**g**) or overflow into the circulation (**b**). The synthesis of noradrenaline from tyrosine occurs in the neuronal cell body and is indicated in grey; catabolic pathways are indicated by dotted arrows. Figure adapted from Gronlund et al. [33]. Abbreviations: CgA, chromogranin a; COMT, catechol-o-methyltransferase; DA, dopamine; DβH, dopamine β hydroxylase; DDC, DOPA decarboxylase; DOPA, dihydroxyphenylalanine; NA, noradrenaline; DHPG, 3,4-dihydroxyphenylglycol; DOPAC, dihydroxyphenylacetic acid; MAO, monoaminoxidase; MHPG, 3-methoxy-4-hydroxyphenylglycol; NMN, normetanephrine; NPY, neuropeptide y; TH, tyrosine hydroxylase; U1, uptake 1; U2, uptake 2; ^3^H, tritium.

Following von Euler’s identification of noradrenaline (sympathin) as the sympathetic neurotransmitter [25] and the subsequent demonstration of an association between the frequency of splenic nerve stimulation and noradrenaline release in the spleen [26], a wide range of measures have been used to provide an index of sympathetic nervous activity in clinical, experimental, and epidemiological studies. While not extensive, as new and apparently novel markers are regularly proposed, the list includes the following:

- Determining the concentration of noradrenaline and metabolites in urine [27], tissue [28], plasma [29] and platelets [30];
- Direct nerve recording using microneurography [31];
- Isotope dilution during a tracer dose infusion of radiolabelled noradrenaline to estimate the rate of release of neurotransmitter (Figure 2) [32];
- Tissue microdialysis [33];
- I123-metaiodobenzylguanidine (MIBG) imaging of the heart [34];
- Assessing the activity and expression of enzymes involved in catecholamine synthesis [35,36];
- Examining catecholamine containing nerve fibres using glyoxylic acid histofluorescence [37];
- Measuring the concentration or immunoreactivity of sympathetic vesicle constituents such as neuropeptide y [38] or chromogranin a [39].

While some of these methods do provide insight into sympathetic nervous processes, whether they provide an accurate indication of sympathetic nervous activity or nerve firing rates is problematic, and interpretation is dependent on the context and consideration of other factors, including rates of synthesis, storage, release, and metabolism of noradrenaline (Figure 2). The tissue concentration of noradrenaline, for instance, provides little information on nerve firing rates. Importantly, levels of noradrenaline in plasma are dependent on the rate of entry into and clearance from the circulation, meaning that elevated plasma levels may arise because of a reduction in plasma clearance rather than an increase in nerve firing. Similarly, the noradrenaline transporter is responsible for the intraneuronal reuptake of noradrenaline [40], meaning that changes in noradrenaline transporter expression may also contribute to alterations in synaptic noradrenaline concentration and influence physiological responses, particularly in the heart, where noradrenaline disposition is largely dependent on neurotransmitter uptake [41]. The expression and trafficking of the noradrenaline transporter to the cell surface is regulated by a raft of intracellular and extracellular compounds, including signalling molecules, acetylcholine, insulin, angiotensin ii, and noradrenaline [42]. Additionally, the expression of the noradrenaline transporter may be reduced due to single-nucleotide polymorphisms [43] and epigenetic mechanisms involving histone modifications on the gene promotor [44]. Preclinical studies have shown that increased plasma glucose and hyperinsulinemia are associated with a reduction in cardiac noradrenaline transporter expression [45], and, in patients with diabetes, cardiac MIBG, which is a target for the noradrenaline transporter, is diminished, being inversely related to the level of haemoglobin a1c [46].

When considering the role of the sympathetic nervous system in the genesis of weight-related physiological complications, it is important to note that sympathetic nervous activity is regionalized and that sympathetic nervous responses are specific to the type and magnitude of the initiating factor or stressor [47]. For instance, the level of change in muscle sympathetic nerve activity (MSNA) during hand grip exercise is dependent on body position and biological sex, with males demonstrating more pronounced increases in MSNA during upright exercise than females [48]. With laboratory mental stress, the response of MSNA demonstrates substantial variability [49]. Similarly, in response to carbohydrates, an elevation of MSNA is evident following glucose but not fructose infusion [50]. Additionally, insulin may drive sympathetic activation via direct central or secondary peripheral vasodilatory (baroreflex) means [51]. While changes in sympathetic nervous activation may precipitate disease development and impact outcomes, it is important to consider the measure in question and any changes in relation to end-organ function and the context in which variations occur.

## 3. Sympathetic Activity and Body Weight

### 3.1. Obesity and Weight Gain

Numerous preclinical studies have documented the effects of weight gain on sympathetic nervous activity. In high-fat-fed rabbits, for instance, the resulting weight gain leads to a rapid increase in renal sympathetic nerve activity [5]. However, in humans, due to the invasive nature of testing, the effects of weight gain on sympathetic drive have been less documented. Nevertheless, work performed by Gentile et al. demonstrated that even modest diet-induced weight gain was accompanied by increased sympathetic neural activity of ∼15–20% in healthy, non-obese males. The increment in MSNA was correlated with the magnitude of body weight and fat gain and was accompanied by increased blood pressure [15]. Similarly, weight gain during late pregnancy has recently been shown to be associated with increased MSNA and higher blood pressure [52]. Cross-sectional studies examining sympathetic tone in individuals with various levels of adiposity are more common. On balance, studies have demonstrated an association between BMI and MSNA in normal-weight and overweight individuals [53]. However, it is important to note that several factors may influence the relationship between adiposity and MSNA. In those with the metabolic syndrome, the elevated MSNA is more strongly associated with blood pressure rather than the degree of obesity [54]. The pattern of sympathetic activation in lean and obesity-related hypertension differs with regard to the firing characteristics of individual sympathetic fibres and the sympathetic outflows involved [55,56]. This implies that the central nervous system drivers of sympathetic activation and potential therapeutic targets differ between the hypertensive phenotypes.

In young adults, we found an association between MSNA and markers of obesity, endothelial dysfunction, and the plasma lipidomic profile [57,58]. In women with polycystic ovary syndrome (PCOS), MSNA was elevated but the degree of activation was not related to BMI [59,60]; rather, circulating androgens were more closely linked to the elevated MSNA [59]. Lansdown and colleagues provided further support for increased MSNA in PCOS and demonstrated an association between functional magnetic resonance imaging (fMRI)-detected activation in the right orbitofrontal cortex and insulin sensitivity [61].

While these observational and experimental studies have demonstrated the detrimental effects of weight gain in adulthood in increasing cardiometabolic risk, it is important to appreciate that obesity and other risk factors in childhood are associated with cardiovascular events later in life [62,63]. Obesity exerts a detrimental effect on cardiovascular structure and function during childhood and adolescence [62] and is a primary correlate of cardiovascular risk factors such as lipids, glucose metabolism, and blood pressure [64]. Although these observations parallel what is seen in adults, whether sympathetic nervous activation plays a role in the complications of obesity in children is difficult to determine, given that few studies have used direct measures of sympathetic activity in this cohort. Resting heart rate has been proposed as a marker of cardiac sympathetic activity [65]. A review and meta-analysis of prospective studies found a positive association between resting heart rate and risk of cardiovascular disease and total cancer and all-cause mortality, with a linear dose–response increase in the relative risk of 15% for cardiovascular disease and 17% for all-cause mortality for each 10 beats per minute increase in resting heart rate [66]. In obese children and adolescents, heart rate has been associated with dyslipidaemia [67] and, across a range of BMI values, with blood pressure and waist circumference [68]. An increased heart rate is not uncommon in obese adults [69] and has been linked to markers of inflammation [70]. Obesity is an important risk factor for the development of obstructive sleep apnoea and, in adults, is characterised by elevated MSNA [71]. Similarly, urinary noradrenaline levels are elevated in children with obstructive sleep apnoea and sleep-disordered breathing [72,73,74], although whether these are linked to obesity is uncertain [72].

A growing body of evidence supports the developmental hypothesis of disease whereby in utero growth restriction and a low birth weight with subsequent accelerated weight gain lead to the development of metabolic disturbances later in life [75]. A low birth weight and accelerated postnatal growth are associated with higher blood pressure [76]. Bhargava and colleagues demonstrated an association between low weight at two years of age and impaired glucose tolerance or diabetes in young adulthood, despite not being obese [77]. Data from the Bogalusa heart study showed an inverse relationship between birth weight and metabolic profile, with a low birth weight being inversely associated with subsequent insulin resistance and elevated plasma triglycerides and total cholesterol in adulthood [78]. Denton and colleagues have similarly described gender-specific differences in sensitivity to changes in the in utero environment, with female offspring being more susceptible to the development of hypertension [79]. Whether these observations are linked to an elevated [80] or reduced [81] sympathetic tone remains unknown.

### 3.2. Weight Loss

#### 3.2.1. Lifestyle—Diet and Exercise

Lifestyle interventions including diet and exercise remain at the forefront in managing and preventing obesity and obesity-related disease. Data from the 2001–2006 National Health and Nutrition Examination Survey indicated that around two-thirds of obese adults had reported trying to lose weight in the previous year [82]. Of these, 40% lost ≥5% and 20% lost ≥10% weight. Successful weight loss was associated with a reduced intake of dietary fat, greater engagement in exercise, the use of prescription weight loss medications, or participation in commercial weight loss programmes [82]. Previous studies have demonstrated the effect of diet-induced weight loss on reducing sympathetic tone in normotensive individuals with obesity [83] or in those with metabolic syndrome [84]. The Dietary Approaches to Stop Hypertension (DASH) diet comprises whole grains, fruits, vegetables, low-fat dairy items, reduced saturated fat, and sodium restriction. Adherence to the DASH diet has been shown to improve metabolic disturbances in patients with type 2 diabetes [85] and reduce sympathetic activity and improve blood pressure, lipids, and insulin sensitivity in individuals with metabolic syndrome [86]. Other dietary interventions using prebiotics to promote short-chain fatty acid production by the gut microbiota hold promise for lowering blood pressure [87]. Similarly, preclinical studies provide some support for the use of plant polysaccharides for modifying metabolic disturbances associated with weight gain [88]. Whether these dietary interventions modify sympathetic nervous activity remains to be determined. Exercise programmes have been shown to reduce MSNA and the whole-body spillover of noradrenaline into plasma [86]. Although exercise programmes are associated with improvement in fitness, body composition, blood pressure, and metabolic profile, they do not necessarily confer any added benefit in reducing sympathetic tone compared to diet alone [86]. Additionally, while exercise is an unequivocally important driver of health, exercise programmes per se provide only a modest benefit in weight loss [89,90].

#### 3.2.2. Bariatric Surgery

Bariatric–metabolic surgery is an important treatment in the management of patients with clinical obesity [4]. Data from the Swedish Obese Subjects study demonstrated that a maximal weight loss between 20 and 32% was achieved 1–2 years post-surgery [91]. The weight loss achieved with surgery has been shown to confer health benefits, including reduced mortality associated with cardiovascular disease and cancer and improved physical and mental health [92]. Ten percent weight loss after laparoscopic adjustable gastric band surgery was accompanied by a reduction in MSNA and improvements in blood pressure, baroreflex sensitivity, metabolic profile, and renal function [93]. Similarly, improvements in metabolic and hemodynamic function and a decrease in MSNA were evident following weight loss after vertical sleeve gastrectomy [94].

#### 3.2.3. Anorexia Nervosa

In women with current anorexia nervosa and a BMI value around 16 kg/m2, MSNA and sudomotor function, as well as systolic blood pressure and heart rate, were significantly reduced compared to healthy counterparts [95]. Decreased plasma noradrenaline and reduced urinary 3-methoxy-4-hydroxyphenylglycol, the deaminated and O-methylated metabolite of noradrenaline (Figure 2), have also been described in acutely ill patients [96]. Depressed sympathetic activity in anorexia nervosa may be attributed to abnormal glucose regulation and low leptin [95]. Anorexia nervosa is known to have a high mortality rate, with a significant contributor of mortality comprising cardiovascular complications encompassing structural, conduction, and haemodynamic abnormalities occurring both during the state of starvation and after treatment with re-feeding [97]. Previous data from Zamboni and colleagues demonstrated that adults with current anorexia nervosa had a higher proportion of visceral than subcutaneous adipose tissue [98], and, at least in the initial period following weight restoration, subjects presented with increased abdominal obesity [99,100], the normalisation of leptin [101], and an increase in MSNA and urinary noradrenaline, albeit to a resting level that remained lower than that seen in healthy BMI-matched control subjects [95,96]. Small increments in abdominal obesity in weight-recovered patients are, however, associated with decreased insulin sensitivity [102]. Whether changes in glucose metabolism and/or leptin concentrations are responsible for sympathetic activation following re-feeding remains unclear.

#### 3.2.4. Cachexia

Cachexia is a hypermetabolic condition characterised by unintended weight loss, muscle and adipose tissue atrophy, systemic inflammation, fatigue, and loss of appetite [103]. It develops in the advanced stages of chronic diseases, primarily in cancer and heart failure, kidney disease, and obstructive pulmonary disease, and is usually associated with poorer prognosis. In cancer-associated cachexia, the browning of white and beige adipocytes accelerates disease progression by increasing sympathetic nervous-stimulated thermogenesis [104]. Additionally, as shown in patients with advanced colorectal cancer, there is a substantial reduction in muscle and fat tissue, with a marked shift in energy expenditure towards high-metabolic tissue such as the liver and metastasis [105]. Given the presentation of the disease and the important role of the sympathetic nervous system in energy disposition, therapeutic targets in cachexia include modifying adrenergic signalling, through agents including, but not limited to, beta-blockers acting in the periphery [103] or centrally acting sympatho-inhibitory agents.

Recent data from the ROMANA 1 and ROMANA 2 clinical trials showed that treatment with the selective ghrelin receptor agonist, anamorelin, was associated with significant weight gain and improved body composition in patients with inoperable lung cancer and cachexia [106]. In patients with heart failure where high sympathetic tone predicted poor prognosis [107], it has been shown that MSNA was higher in those who experienced weight loss and was the best predictor of subsequent unintentional weight loss in this population [108]. This suggests that reducing sympathetic tone may be a target in preventing weight loss in patients with heart failure.

#### 3.2.5. Pharmacological Agents

Pharmacological interventions may be needed to augment the management of body weight and improve weight-related co-morbidities such as insulin resistance, glucose tolerance, dyslipidaemia, and hypertension. Previous reports have reviewed drug classes associated with weight management and their influence on end-organ function [109]. While in some earlier studies, beta adrenergic blocking drugs were shown to promote weight gain and worsen insulin resistance, newer agents, such as carvedilol and nebivolol, may exert a more favourable effect on metabolic parameters [110,111,112]. Similarly, centrally acting agents such as imidazoline I1 agonists may improve metabolic control [113]. Here, we concentrate on some of the recent developments, with a focus on incretins and glifozins. Pharmacological approaches to weight gain in cachexia and anorexia nervosa have been noted previously.

##### Incretins

The incretins, glucagon-like peptide-1 (GLP-1, produced by the L cells of the lower gut) and glucose-dependent insulinotropic peptide (GIP, produced by the K cells of the upper gut), are released shortly after eating to stimulate insulin and glucagon secretion. Both incretins are rapidly deactivated by dipeptidyl peptidase 4 (DPP4). Functionally, the L cells in the gut are innervated by sympathetic nerves, which, if activated, suppress postprandial GLP-1 secretion and impair glucose utilisation [114]. Receptors for GLP-1 are distributed throughout the body and may exert effects beyond glucose control and satiety [115]. For instance, Pauza et al. demonstrated GLP-1 receptor expression in the carotid body in rats and humans and found that its decreased expression was linked to sympathetic activation, driving blood pressure elevation in obese rats [116].

An increasing number of GLP-1 agents have been developed and trialled, showing improvement in glucose control and weight reduction [117]. The recent demonstration of the effectiveness of tirzepatide [118] and retatrutide [119] holds promise for combination therapy targeting GLP-1, GIP, and glucagon to promote weight loss and reduce obesity-related co-morbidities. These agents exert beneficial effects in addition to weight loss, with data from the LEADER Trial indicating that the use of the GLP-1 receptor agonist, liraglutide, reduced death from cardiovascular disease, nonfatal myocardial infarction, and nonfatal stroke among patients with type 2 diabetes [120]. Further, tirzepatide, which is a GIP and GLP-1 receptor agonist, improved blood pressure and physical aspects of quality of life [118], and the triple combination, retatrutide, reduced liver and abdominal fat and improved insulin sensitivity and lipid metabolism in obese individuals [121]. Paradoxically, given the relationship between heart rate and mortality [66], these improvements following therapy contrast with the effect of GLP-1 agonists on heart rate, with short-acting drugs associated with a transient increase of 1–3 beats per minute and longer-acting agents associated with a rise of around 3–10 beats per minute [122]. The increased heart rate following GLP-1 therapy likely does not engage the autonomic nervous system, perhaps explaining this paradox. In a comprehensive series of experiments, Lubberding and colleagues demonstrated that the chronotropic effect of GLP-1 is independent of sympathetic and vagal innervation; rather, it depends on the direct activation of GLP-1 receptors in pacemaker cells of the sinus node [123]. At least acutely, in humans, GLP-1 administration has been shown to exert a differential effect on cardiac and muscle vasoconstrictor sympathetic outflows, increasing MSNA with no change in heart rate or measures of heart rate variability [124].

##### Glifozins

Sodium glucose cotransporters (SGLTs) occur mainly in the kidneys and play a key role in managing hyperglycaemia independently of insulin [125]. By inhibiting the SGLT2 receptor, gliflozins prevent the reuptake of glucose by the kidney and lower the glucose level in the blood by promoting the excretion of glucose in the urine. Many trials have demonstrated the benefit of SGLT blockade, with a recent meta-analysis clearly showing that SGLT2 inhibition reduces the risk of major adverse cardiovascular events across patient groups, irrespective of the presence of atherosclerotic cardiovascular disease, diabetes, or renal function at baseline [126]. The effect was evident largely due to a reduction in cardiovascular death, particularly heart failure and sudden cardiac death. A further meta-analysis of trials revealed that patients receiving an SGLT2 inhibitor of combined SGLT1/SGLT2 inhibitors had a mean body weight reduction of −1.79 kg (BMI change −0.71) compared with the placebo [127].

Indicative of the effect of SGLT receptor blockade on sympathetic innervation, kidneys and hearts from neurogenic hypertensive Schlager mice treated with dapagliflozin displayed reduced tyrosine hydroxylase staining and a reduced kidney noradrenaline content compared with control mice [128]. In parallel, sympathetic denervation with 6-hydroxydopamine reduced blood pressure, improved glucose metabolism, and led to a reduction in SGLT2 expression in the kidney [128]. A role for SGLT receptors in regulating sympathetic outflow is further supported by the demonstration of SGLT1 and SGLT2 receptors in the RVLM in the brainstem [129] and SGLT2 receptors in the hypothalamus, amygdala, and periaqueductal grey [130] of the mouse. Treatment with the SGLT2 receptor blocker, empagliflozin, hyperpolarized neurones in the RVLM [129], thereby providing evidence of potential central sympatho-inhibition associated with SGLT receptor blockade. Treatment with dapagliflozin was associated with increased c-fos expression in the hypothalamus, amygdala, and periaqueductal grey [130]. While these experiments provide some support for interaction between SGLT receptor blockade and sympathetic nervous activity, short-term treatment with empagliflozin in patients with type 2 diabetes had no effect on MSNA [131]. In a more recent study from the same group, Heusser and colleagues observed no difference in MSNA between treatment with an SGLT2 blocker and hydrochlorothiazide in patients with type 2 diabetes [132]. The change in MSNA following therapy was negatively associated with the change in body weight, with empagliflozin treatment showing a greater weight loss [132]. The authors proposed that the changes observed may have been influenced by changes in renal sodium excretion. Whether SGLT blockade influences cardiac or renal sympathetic activity in humans remains unknown.

## 4. Brain Pathways Associated with Sympathetic Regulation

**Figure 3:**
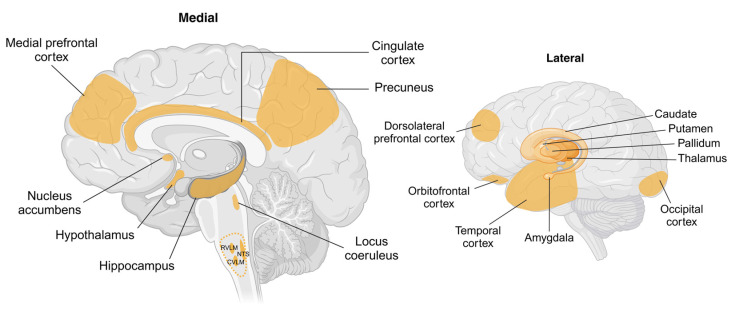
Schematic showing brain regions associated with changes in body weight and sympathetic nervous system activity. Key cortical and subcortical regions within the brain undergo structural and functional changes with weight gain or weight loss. Hypothalamic regions including arcuate, paraventricular, ventromedial, and dorsomedial nuclei are identified as targets for peripherally or centrally originating afferent and humoral signals involving adipokines, insulin, and glucose. Key brainstem regions including the nucleus tractus solitarius (NTS) and caudal and rostral ventrolateral medulla (CVLM and RVLM, respectively) are recognised as being involved in the generation of sympathetic nerve activity, with RVLM being the primary nucleus for outflow to renal, splanchnic, and muscle vascular beds. The cortical regions shown are known to be influenced by body weight; however, the pathways by which this occurs are not well understood. Abbreviations: CVLM, caudal ventrolateral medulla; NTS, nucleus tractus solitarius; RVLM, rostral ventrolateral medulla. Created in BioRender. Braun, J. (2025) https://BioRender.com/a80q797, accessed on 18 March 2025.

While the sympathetic nervous system is, by definition, part of the peripheral nervous system, it is regulated by a coordinated interplay between peripheral and central processes. Direct monosynaptic inputs from the brain to the sympathetic preganglionic neurones in the intermediolateral column (Figure 3) arise from the RVLM, caudal ventrolateral medulla (CVLM), paraventricular nucleus (PVN) of the hypothalamus, caudal raphe, and pontine noradrenergic A5 group [133]. Work in animals has demonstrated the importance of the RVLM, along with its connections with the brainstem and hypothalamic nuclei, in regulating sympathetic outflow to the muscle, splanchnic, and renal vascular beds [134,135]. The beat-to-beat regulation of sympathetic tone is achieved through the coordinated activity of the nucleus tractus solitarius (NTS), which receives baroreceptor input and projects excitatory signals to the CVLM, which, in turn, leads to the inhibitory control of the RVLM [134]. Similarly, pressor noradrenergic neurones in the A5 region, locus coeruleus (A6 region), and hypothalamus have been shown to influence sympathetic outflow [136,137,138]. Experimental studies using pseudorabies virus-mapping techniques identified neuronal groups in the PVN, the arcuate nucleus (ARC), and the NTS and A5 noradrenergic group as having direct connections with the liver and white adipose tissue [139]. Studies in humans using internal jugular vein blood sampling provided some evidence for an association between brain noradrenaline turnover, changes in pulmonary wedge pressure, and cardiac sympathetic activity [140], whilst the internal jugular venous overflow of noradrenaline provides an index of cerebrovascular sympathetic nerve activity [141]. Interestingly, most noradrenaline in the brain is in the locus coeruleus [142], which, recently, through its pulsatile release of noradrenaline, has been shown to influence the cerebral vasculature and play a pivotal role in the regulation of the glymphatic system during sleep [143]. Studies in humans utilising direct recordings of MSNA provide us with a picture of the near-real-time correlation of sympathetic outflow with activity in the brain. Macefield and Henderson coupled fMRI with MSNA and identified several cortical and subcortical brain regions that contribute to central autonomic control [144,145]. Further, signal intensity within key hypothalamic nuclei including the ventromedial hypothalamus (VMH) and dorsomedial hypothalamus (DMH) have also shown significant positive covariations with MSNA [146]. The ARC within the hypothalamus is known to have neuronal projections to the VMH and DMH but was not reported to have significant associations with MSNA. Overall, in the cortex, regions including the insula, dorsolateral prefrontal cortex, posterior cingulate, and precuneus also showed positive correlations with MSNA. Further, the same study also reported the functional connectivity of the RVLM with the VMH and DMH, as well as the thalamus, insula, posterior cingulate, and precuneus. Similarly, the VMH was shown to be functionally coupled to the RVLM, insula, dorsolateral prefrontal cortex, and precuneus, highlighting the complexity of sympathetic regulation and the contribution of these cortical and subcortical regions to the sympathetic connectome.

### 4.1. Brain Pathways Associated with Weight Gain

Preclinical and clinical investigations using neuroimaging techniques such as magnetic resonance imaging (MRI), fMRI, positron emission tomography (PET), computed tomography (CT), and magnetoencephalography (MEG) have been used to gain insight into the key brain regions that undergo structural and functional changes with weight gain. Although several articles have discussed obesity- and weight-loss associated brain changes in the context of reward circuitry, cognitive function, and energy metabolism [147,148,149,150,151], here, we summarise obesity-associated changes in brain function with a particular focus on regions linked with sympathetic dysregulation.

Tadross and colleagues recently published a comprehensive cellular transcriptomic map of the human hypothalamus, highlighting spatially distinct neuronal clusters and noting that six genes (MC4R, PCSK1, POMC, CALCR, BSN, and CORO1A) were associated with BMI at the population level [152]. Not surprisingly, some of these genes are prominent in pathways involved in the regulation of satiety and energy balance and may influence sympathetic nervous regulation. For instance, increased adiposity drives hyperleptinemia. The elevated circulating leptin acts on the proopiomelanocortin (POMC) neurones in the ARC and agouti-related peptide (AgRP) neurones, leading to ARC-specific leptin resistance [153]. In the lean state, the leptin-induced activation of POMC neurones in the ARC activates melanocortin receptor neurones in the PVN and DMH, thereby increasing sympathetic outflow to BAT [154]. The microinjection of leptin within the VMH has been shown to increase renal sympathetic nerve activity and blood pressure in rats [155]. Additionally, studies by Greenwood and colleagues have provided support for melanocortin signalling in the control of human blood pressure through an insulin-independent mechanism [156]. The melanocortin-receptor-expressing neurones of the VMH were shown to receive input from the POMC and AgRP neurones of the ARC, and the activation of POMC neuronal input to the VMH was demonstrated to increase renal sympathetic nerve activity in obese rabbits [157]. The neuronal populations within the ARC and VMH are also sensitive to circulating glucose [158]. Obesity-associated neuroinflammation, particularly in the hypothalamic neurones and glial cells, may drive glucose intolerance and insulin resistance [159].

Regions within the brainstem and associated hypothalamic nuclei play a role in the beat-to-beat generation of sympathetic outflow [135]. Studies in obese Zucker rats have shown that impaired glutamatergic activation in the NTS is associated with increased sympathetic nervous activity and impaired baroreflex control [160]. Previous studies in animals showed that a high-fat diet promoted oxidative stress and inflammation within the RVLM which, combined, contributed to obesity-induced sympatho-excitation [161]. In line with this evidence, therapeutic interventions such as calorie restriction, which produces an anti-oxidant effect in the RVLM [162], results in weight loss and inhibits sympathetic drive. Neural activity in subcortical regions including the amygdala and hippocampus has also been shown to be associated with BMI [163]. While cortical regions undergo structural and functional changes in obesity, whether they are associated directly with changes in sympathetic activity remains unknown.

### 4.2. Brain Pathways Associated with Weight Loss

Approaches to intentional weight loss may include lifestyle changes such as diet and exercise, bariatric surgery, and pharmacological interventions. The changes in brain activity and connectivity associated with these interventions have recently been reviewed [151]. With bariatric surgery, the reduction in plasma ghrelin that occurs following sleeve gastrectomy was associated with decreased activation in the brain’s reward-related areas in response to energy-rich foods [164]. In line with this observation, the intravenous infusion of ghrelin in lean and overweight individuals, at least acutely, has been shown to increase MSNA and plasma cortisol [165]. Improvements in both grey and white matter volumes and functional connectivity across multiple brain regions following sleeve gastrectomy have also been described [166]. Two years following Roux-en-Y surgery, improvements in cognition and in cardiovascular and mental health were accompanied by increased cortical thickness and maintenance of white matter and hippocampal volume [167]. Changes in brain function following lifestyle or pharmacological interventions depend, at least in part, on the intervention (for a review, see [151]) and the success in attaining weight loss. For instance, changes in the mesolimbic reward centre were related to reduced ghrelin levels and were only evident in those individuals who had achieved successful weight loss [168]. With GLP-1 receptor agonist-induced weight loss, gut-derived signals stimulate the NTS, nucleus accumbens, and ventral tegmental area, increasing serotonergic activity to promote satiety [169]. Additionally, the activation of GLP-1 receptors in the area postrema in the brainstem modulates dopaminergic activity to reduce food intake and modulate activity in the ARC of the hypothalamus [170,171]. With unintentional weight loss, as occurs in, for instance, cancer cachexia, alterations in the gut microbiome have been shown to drive hypothalamic inflammation and changes in PVN activity in an animal model [172]. In patients with anorexia nervosa, a widespread reduction in cortical thickness and reduced volumes in subcortical structures including the nucleus accumbens, amygdala, caudate, pallidum, hippocampus, putamen, and thalamus have been described [173]. Importantly, the degree of change in brain structure was associated with BMI and was improved in those with partial weight recovery.

In summary, the monosynaptic (direct) determinants of sympathetic outflow from the intermediolateral column in the spinal cord are well described. It is important to note that while these regions are important in the regulation of sympathetic nervous activity, higher brain regions are also engaged and respond to a wide range of humoral, hormonal, neural, and psychological stimuli. The diversity of inputs controlling sympathetic outflow ensures that sympathetic responses are regionalized and likely offers precision and gain in regulating efferent responses. The brain regions implicated in the context of sympathetic activity and weight change include the ARC, PVN, VMH, and DMH within the hypothalamus and RVLM, A5 group, and NTS within the brainstem. These regions receive afferent and humoral inputs that influence sympathetic outflow to various effector organs. Cortical areas including the frontal, temporal, occipital, and insular regions have also been shown to undergo structural and functional changes associated with increased adiposity; however, further investigations are required to fully understand the mechanisms driving these changes and their relationship with sympathetic activity. Importantly, changes in sympathetic activity and end organ function may occur through direct actions via the stimulation of the sympathetic regulatory centres or secondarily to reflex, hormonal, or paracrine signalling.

## 5. Regional Sympathetic Nervous Activity and Cardiometabolic Risk

**Figure 4:**
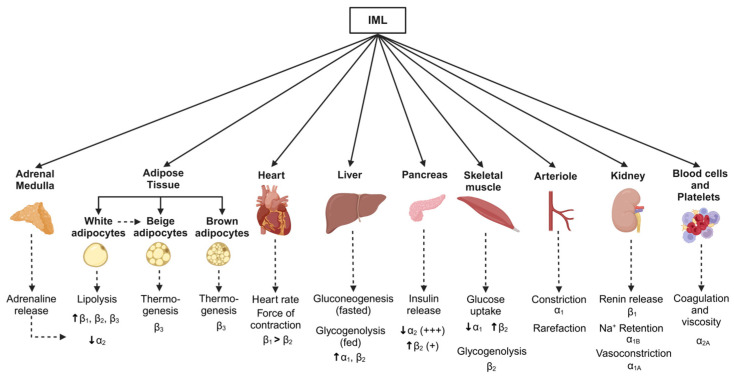
Schematic showing metabolic pathways associated with changes in body weight and sympathetic nervous system activity. Monosynaptic projections arising from brain regions including the rostral and caudal ventrolateral medulla, caudal raphe, and pontine noradrenergic A5 group, as well as the paraventricular nucleus of the hypothalamus, provide inputs to the sympathetic preganglionic neurones in the intermediolateral cell (IML) column of the spinal cord. Sympathetic postganglionic neurones then project them to target organs (solid arrows). Although sympathetic nervous-associated pathways vary between the weight-gain and weight-loss states, key effector organs and the corresponding adrenergic receptors in response to sympathetic activation are shown (dotted arrows). Created in BioRender. Braun, J. (2025) https://BioRender.com/t56w833, accessed on 18 March 2025.

The global escalation in obesity has led to a pronounced increase in the prevalence of metabolic syndrome, a clustering of metabolic disturbances and cardiovascular risk factors that is characterised by abdominal obesity, hyperglycaemia, impaired insulin sensitivity, high blood pressure, abnormal lipids, and a systemic pro-inflammatory state. Obesity contributes to cardiovascular risk factors, and, additionally, exerts direct adverse effects on cardiac structure and function [174], contributing to the development of both atherosclerotic and non-atherosclerotic cardiovascular disease independently of other risk factors [175]. While the pattern of cardiometabolic risk associated with weight gain and obesity has been well described, the role of the sympathetic nervous system in generating or modifying risk is likely dependent on the pattern and strength of the sympathetic outflows engaged (Figure 4). While intentional weight loss exerts positive effects on sympathetic tone, unintentional weight loss, as occurs in anorexia nervosa or cachexia, is associated with detrimental physiological effects. In this section, we review the influence of weight change on regional sympathetic activity. It is important to note that effects on an organ may influence other vascular beds, and so effects should not necessarily be considered in isolation but, rather, considered as a network of integrated activities.

### 5.1. Adipose Tissue

Obesity and the expansion in fat mass that typically occurs with weight gain are associated with sympathetic nervous activation [53] and increased cardiometabolic disease risk [176]. Data show that the type and distribution of fat are important determinants of disease development, with visceral obesity being the prime driver of sympathetic nervous activation [15,177,178]. Recent data point to a role of intramuscular fat as an important determinant of cardiometabolic disease development and progression [179]. Muscle sympathetic nerve activity is elevated in men with visceral but not subcutaneous obesity [177], and even modest increases in adipose tissue mass are linked with increased MSNA [15]. The classification and function of adipose tissue is dependent on the type of adipocytes present. White adipose tissue (WAT) is principally involved in energy storage and BAT in thermogenesis. Beige adipocytes have thermogenic properties and are found within WAT depots [180]. White adipose tissue is distributed throughout the body and forms most subcutaneous, visceral, and perivascular fat depots. The adipocytes in WAT store energy in the form of triglycerides loaded in large lipid droplets [181]. Energy production occurs through the generation of free fatty acids (FFAs) via lipolysis through the actions of insulin, the sympathetic nervous system, and catecholamines via action on adrenergic receptors [182]. Conversely, increasing sympathetic drive to BAT facilitates energy expenditure and the generation of heat through the stimulation of BAT thermogenesis [183].

The detection of BAT in adult humans [184] has raised the possibility that dysfunction in the genesis or function of BAT may underpin, and predispose someone to, the development of obesity. BAT activity, which is under sympathetic control [185], can be assessed by combining single-photon emission CT (SPECT)/CT and 18 F-flurodeoxyglucose PET/CT scanning [186]. While observations indicating a negative association between the amount of BAT and BMI [184], central obesity and liver fat [187], and altered function in obesity [13,14] indicate a possibly important role for BAT in metabolism and weight maintenance, the actual mechanisms at play remain to be determined. Carey and colleagues demonstrated that ephedrine could activate BAT in lean but not obese individuals [14], whereas Bahler et al. found no difference in BAT activity in response to cold exposure between lean and obese men [188]. Recent studies using the β3 receptor agonist, mirabegron, demonstrated no effect on body weight but an improvement in glucose tolerance, haemoglobin A1c, and insulin sensitivity in obese subjects after 12 weeks of treatment [189].

Leptin is produced predominantly by fat cells [190], with subcutaneous fat being the main site of leptin production [191]. The concentration of leptin in plasma and cerebrospinal fluid is proportional to the degree of adiposity [192]. As noted previously, leptin released into the circulation acts centrally to influence appetite and energy expenditure, but in the obese state, the metabolic action of leptin is impaired [193], whilst its sympathetic cardiovascular stimulatory effect remains preserved [194]. Studies in rabbits exposed to a high-fat diet have shown that leptin plays a role in driving obesity and hypertension via a stimulatory effect on renal sympathetic nerve activity [5]. Whether this is the case clinically remains uncertain. In human obesity-related hypertension, there is evidence of sympathetic activation, based on an almost two-fold increase in the rate of spillover into plasma of noradrenaline in the renal veins [195] and increased nerve firing to the skeletal muscle vascular bed [196].

Adipose tissue plays an important role in energy storage and metabolism. Triacylglycerol stored in adipose tissue can be rapidly metabolised, converted to NEFAs, and released into the circulation for use as an energy source [197]. Meanwhile, white adipose tissue is innervated by sympathetic nerves, and sympathetic activation to white fat is essential for lipolysis [183]. Previous work by our group has noted associations between MSNA and elements of the circulating lipidomic profile, including NEFAs and triacylglycerol [58]. Circulating FFAs in plasma are an energy substrate and influence the development of insulin resistance. Free fatty acid availability is determined mainly by the rate of mobilisation from adipose tissue triacylglycerol stores via the direct effect of sympathetic activation on lipolysis [183]. Other factors such as insulin, growth hormone, natriuretic peptides, and some adipocytokines may also influence lipolysis. Free fatty acids can act throughout the body to disrupt insulin signalling and impair glucose uptake. In obesity, the increase in circulating FFAs that occurs with increased lipolysis contributes to the development of insulin resistance, glucose intolerance, and hypertension through combined effects on skeletal muscle and the renin–angiotensin system [197].

Adipose tissue is also the site of the production and release of innate and adaptive immune cells and numerous cytokines, including leptin, adiponectin, resistin, tumour necrosis factor alpha, and interleukin-6. Through their various pro- and anti-inflammatory processes, adipokines can exert positive or negative effects on a range of conditions including insulin resistance, diabetes, inflammation, and atherosclerosis [7]. An increase in the expression of pro-inflammatory monocytes occurs in obesity, with their abundance being associated with an increasing BMI and waist circumference, elevated circulating triglycerides and haemoglobin A1c, and decreased high-density lipoprotein (HDL)-cholesterol [198]. Immune cells express adrenoceptors on their cell surface [199], raising the possibility that sympathetic activation contributes to adipose tissue inflammation [199]. Dietary interventions can exert differential effects in the production of anti-inflammatory adipokines, such as adiponectin, and pro-inflammatory adipokines, including interleukin-6 [200], and positively influence endothelial function [200] and measures of whole-body noradrenaline spillover into plasma and MSNA [84].

### 5.2. Liver and Pancreas

Noradrenaline-containing nerve fibres arise from the celiac and superior mesenteric ganglia and project to hepatocytes in the liver [201]. The stimulation of the hepatic nerve in humans causes a rapid increase in circulating glucose [202]. Similarly, the release of catecholamines following stimulation of the adrenal medulla is associated with hepatic glucose production [203], although with stress-induced hyperglycaemia, this occurs subsequently to the initial hypothalamic-induced sympatho-excitation [204]. The sympathetic nervous system promotes glucose production by activating glycogen breakdown (glycogenolysis) after a meal and increasing gluconeogenesis (glucose production from lactate, pyruvate, and fatty acids) during overnight fasting or between meals. Previous work by Cherrington and colleagues has demonstrated the important regulatory role of the sympathetic innervation of the liver in the uptake of glucose [23]. Indeed, the liver plays an important role in mediating glucose homeostasis following a meal. In healthy individuals, this is achieved by reducing hepatic glucogenesis and stimulating hepatic glucose uptake [23]. Sympathetic nervous activation in the liver inhibits hepatic glucose uptake [24]. Experimental data in dogs have shown that an increased concentration of glucose in the portal vein initiates the uptake and storage of glucose by the liver, with the effect being blunted by a high-fat, high-fructose diet [205]. In obese individuals and in patients with type 2 diabetes, the rates of hepatic gluconeogenesis and glycogenolysis are increased and the rates of glycogen synthesis are decreased, which, combined, leads to elevated fasting and postprandial glucose levels [206]. Indeed, the activation of the sympathetic nervous system is evident along the diabetes continuum, with elevated sympathetic activity occurring in subjects with impaired glucose tolerance [207] and being further augmented in patients with type 2 diabetes [207,208]. Kato et al. demonstrated that steatosis was linked with raised insulin resistance in the muscle in patients with non-alcoholic fatty liver disease [209]. In an animal model, diet-induced steatosis was associated with a two-fold increase in the firing rate of the hepatic sympathetic nerves [210]. The subsequent denervation of the hepatic sympathetic nerves improved steatosis and liver triglyceride accumulation, independent of changes in body weight, caloric intake, or body fat [210]. Taken together, these data clearly demonstrate an important role of the hepatic sympathetic nerves in generating at least some of the complications associated with the development of non-alcoholic fatty liver disease.

The pancreas is composed of multiple cell types including alpha (producing glucagon), beta (producing insulin), delta (producing somatostatin), and pancreatic polypeptide-producing cells. While the beta and alpha cells in the pancreas respond directly to changes in blood glucose, the neural input from the autonomic nervous system also plays an important role in glucose regulation. In general, sympathetic activation to the pancreas increases blood glucose through a combined effect on alpha and beta cells, increasing glucagon and reducing insulin secretion [211]. Additionally, sympathetic nerves have been shown to innervate endothelial cells adjacent to capillaries and reduce capillary diameter and islet blood flow in pancreatic slices [212,213]. Taken together, these observations demonstrate the multiple mechanisms by which the autonomic nervous system can influence glucose disposition through direct or indirect effects on the pancreas.

In line with clinical observations [15,207], a recent experimental study reinforced that diet-induced insulin resistance and metabolic disorder occur through increased sympathetic nervous system activity [214]. The sympathetic stimulatory effects of insulin are thought to be mediated by a direct increase in centrally driven sympathetic nervous outflow and a baroreflex-mediated response to the vasodilatory actions of insulin [51]. Work by Sakamoto and colleagues showed in a mouse model that reduction in catecholamine signalling reduced lipolysis and protected against diet-induced insulin resistance, hyperglucagonemia, adipose tissue dysfunction, and the development of fatty liver disease [214].

### 5.3. Skeletal Muscle

Previous studies have clearly demonstrated that the sympathetic nervous system has anabolic effects on skeletal muscle [215] and that skeletal muscle is responsible for the majority of glucose uptake following an oral glucose load [216]. The development of insulin resistance is associated with the desensitisation of muscle to insulin released by the pancreas to drive glucose uptake, resulting in elevated levels of circulating glucose [216]. Skeletal muscle insulin resistance can, in fact, appear well before the onset of pancreatic β-cell failure and the diagnosis of type 2 diabetes. Sympathetic nerves innervating skeletal muscle can modulate glucose uptake via the activation of β-adrenergic receptors. Conversely, the stimulation of α-adrenergic receptors in the vasculature can impair glucose uptake in skeletal muscle via an effect on glucose delivery. While the sympathetic nervous innervation of blood vessels supplying skeletal muscle is well recognised [217], recent studies have also demonstrated a clear role for sympathetic nerves at the neuromuscular junction [218], with sympathetic activity playing an important role in maintaining skeletal muscle structure and function [219]. In a mouse model of heart failure, sympathetic activation was demonstrated to contribute to the morphological alterations in skeletal muscle myopathy [220]. In patients with heart failure and sarcopenia, MSNA was elevated, compared to patients without sarcopenia, and was negatively correlated with appendicular lean muscle mass [221]. With ageing, a selective loss of sympathetic axons projecting to myofibers and the neuromuscular junction occurs, resulting in a degree of muscle denervation [218,222].

### 5.4. Kidney

The increase in the prevalence of obesity has seen a parallel rise in the incidence of chronic kidney disease [223,224]. Importantly, damage to the kidneys, as indicated from the urinary albumin/creatine ratio, is an independent predictor of mortality risk in the general population [225]. Although the impact of obesity on chronic kidney disease may be influenced and accentuated by comorbidities such as diabetes and hypertension, excess weight and visceral fat are crucial in disease development [226]. Recent data derived from the National Health and Nutrition Examination Survey have demonstrated a substantial rise in the prevalence of obesity-related hypertension over the last two decades [227]. Increased renal sympathetic activity is evident in obesity, with elevated noradrenaline spillover into the renal veins evident in both normotensive and hypertensive obese individuals [195]. At a young age, alterations in kidney function are evident with weight gain. Overweight or obese young adults present with elevated creatinine clearance compared with their lean counterparts, with the increment in creatinine clearance being directly and positively correlated with MSNA [228]. Indeed, sympathetic activation to the kidney may contribute to the development of renal diseases by causing glomerular hyperfiltration and injury to the glomerular and proximal tubules [229]. Additionally, obesity-induced compression of the kidney may lead to increased sodium reabsorption and contribute to renal vasodilation, glomerular hyperfiltration, and increased renin secretion in obese subjects [226]. Radiofrequency ablation of the renal sympathetic nerves has been shown to reduce blood pressure in patients with resistant hypertension [230] and improve insulin sensitivity [231], perhaps due to the denervation of renal afferents projecting from the diseased kidney to the brain. While the first-line management of cardiovascular and kidney complications associated with weight gain includes diet and exercise, cessation of tobacco smoking, glycaemic and dyslipidaemia control, and lowering blood pressure, additionally, there are four pharmacological pillars of therapy. These include targeting the renin–angiotensin–aldosterone system, SGLT-2 inhibition, the blockade of the mineralocorticoid receptor, and GLP-1 agonists [232]. Whether the therapeutic benefit of these agents rests solely with their direct pharmacological action or works via a secondary beneficial effect on modulating sympathetic tone is not known.

### 5.5. Heart and Vascular Function

Longitudinal data from the Framingham Heart Study indicated that each 1 kg/m2 increase in BMI over 14 years was associated with a 5–7% increase in the risk of heart failure [233]. Importantly, weight-related changes in left ventricular structure and function are evident even in children [234] and young and middle-aged individuals prior to the onset of clinical signs of disease [228,235]. Indicative of a role of the sympathetic nervous system in the development of cardiac hypertrophy, alterations in left ventricular structure and function have been shown to correlate with MSNA in young overweight subjects [228]. The obesity-related activation of the renin–angiotensin system may also play a role in cardiac remodelling. The release of angiotensin II, combined with the parallel increase in sympathetic nervous activity, may lead to vasoconstriction, fluid retention, and increased left ventricular pre- and afterload [236]. The observations of Burns and colleagues [237], who showed that combined treatment targeting the sympathetic and renin–angiotensin–aldosterone system provided a greater reduction in left ventricular mass compared with a thiazide diuretic and calcium channel blocker, despite equal blood pressure reduction, provided further support for the engagement of the sympathetic nervous and renin–angiotensin–aldosterone systems in generating left ventricular hypertrophy.

Vascular ageing is an important determinant of cardiovascular morbidity and mortality and can be examined using a raft of techniques, including the measurement of endothelial dysfunction, arterial stiffness, and carotid intima-media thickness [238]. Endothelial dysfunction has been shown to precede the development of atherosclerosis and is one of the early factors in the cascade of cardiovascular disease development. Similarly, large-artery stiffness is an independent predictor of all-cause and cardiovascular mortality [239]. Although these techniques are widely used to assess “vascular ageing” in general, the measures likely reflect differing underlying physiology [240]. Previous studies have demonstrated an association between MSNA and endothelial dysfunction in lean [241] and overweight or obese adults [228], with only modest gains in visceral fat mass being shown to be linked with endothelial dysfunction [242]. In line with this, a recent analysis of data derived from the UK Biobank and Fuqing Cohort indicated an association of arterial stiffness with central obesity but not BMI [243]. Oren and colleagues examined aortic stiffness using pulse wave velocity and carotid intima-media thickness and found an association between BMI and carotid intima-media thickness but not BMI and pulse wave velocity, which was related to male sex and blood pressure [240]. The habitual consumption of a diet high in saturated fat in relation to poly-unsaturated and mono-unsaturated fat was strongly associated with impaired endothelial function in young overweight adults [244].

A large body of literature has contributed to our understanding of the mechanisms of vascular ageing and how perturbations initiate the development and worsening prognosis of cardiovascular disease [245,246]. The mechanisms involved include increased adipokine-induced inflammation, hyperinsulinemia, reciprocal changes in low- and high-density lipoproteins, and the activation of the renin–angiotensin–aldosterone systems. While an association between MSNA and endothelial function in young overweight individuals has been described [228,241] and MSNA has been shown to be associated with increased pulse wave velocity in both peripheral and central vessels [247], whether the activation of the sympathetic nervous system contributes to vascular ageing independently of changes in blood pressure or lipids remains to be determined.

## 6. Conclusions

A body of evidence has demonstrated the importance of the sympathetic nervous system in weight regulation and in the cardiometabolic consequences associated with changes in body weight and body composition. Preclinical studies and the advancement and application of brain-mapping techniques in humans have contributed to our understanding of the brain regulatory pathways involved in the regionalization of sympathetic outflow and continue to provide insights into the molecular pathways involved in sympathetic regulation associated with weight changes. Recent developments in treatments for weight gain and weight loss have benefited from these findings, with approaches developed to target pathways and pathophysiology to improve clinical outcomes. Understanding the brain pathways involved in specific sympathetic regulation may help in the development of centrally targeted therapies that reduce the burden of cardiovascular and metabolic decline seen with weight gain. In the era of more personalised medicine, future therapies may include new agents, re-purposed pharmaceuticals, or neuromodulatory approaches involving stimulation or denervation. Advances in electrode development and placement and the use of closed-loop designs hold promise for further advances in the field. Preclinical studies have shown that abdominal vagal nerve stimulation improves intestinal inflammation in the absence of off-target cardiovascular effects [248], and closed-loop approaches have been developed and used for the treatment of epilepsy [249] and diabetes [250]. Interestingly, it has previously been shown that transcutaneous vagal nerve stimulation in the ear is associated with a reduction in MSNA [251]. Device-based therapies to modify sympathetic nervous activity to this juncture have been confined largely to renal [230] and carotid body [252] denervation. Approaches in the future may utilise different approaches to deliver energy and more specific targeting of afferent or efferent nerves or receptors mitigating the physiological response.

Taken together, the available data provide strong evidence of sympathetic nervous activation occurring with weight gain and sympathetic inhibition with intentional weight loss. The degree of activation is, in general, proportional to the excess weight and is strongly linked to the distribution of fat, with intra-abdominal visceral fat being an important driver of sympathetic activation. Additionally, the hepato-portal circulation and related organs receive sympathetic innervation and are pivotal in the control of glucose homeostasis. The increase in sympathetic activity is observed early with weight gain and, if prolonged, is associated with complications, including high blood pressure and structural and functional alteration in the heart, and detrimental changes in circulating lipids and insulin sensitivity. In the current review, we have evaluated the mechanisms and responses associated with sympathetic dysfunction in body weight regulation. Despite the complexity of these underlying mechanisms, the diverse regulatory sites may provide opportunistic targets and approaches for therapy.

## Data Availability

No new data were created or analyzed in this study. Data sharing is not applicable to this article.
